# No evidence for spatial suppression due to across-trial distractor learning in visual search

**DOI:** 10.3758/s13414-023-02667-8

**Published:** 2023-02-23

**Authors:** Ai-Su Li, Louisa Bogaerts, Jan Theeuwes

**Affiliations:** 1 Department of Experimental and Applied Psychology, Vrije Universiteit Amsterdam, Van der Boechorststraat 7-9, 1081 BT Amsterdam, The Netherlands; 2 Institute Brain and Behavior Amsterdam, Amsterdam, the Netherlands; 3 Department of Experimental Psychology, Ghent University, Ghent, Belgium; 4 William James Center for Research, ISPA-Instituto Universitario, Lisbon, Portugal

**Keywords:** Across-trial regularities, Statistical learning, Spatial suppression, Sequence learning, Visual search

## Abstract

Previous studies have shown that during visual search, participants are able to implicitly learn across-trial regularities regarding target locations and use these to improve search performance. The present study asks whether such across-trial visual statistical learning also extends to the location of salient distractors. In Experiments 1 and 2, distractor regularities were paired so that a specific distractor location was 100% predictive of another specific distractor location on the next trial. Unlike previous findings that employed target regularities, the current results show no difference in search times between predictable and unpredictable trials. In Experiments 3–5 the distractor location was presented in a structured order (a sequence) for one group of participants, while it was presented randomly for the other group. Again, there was no learning effect of the across-trial regularities regarding the salient distractor locations. Across five experiments, we demonstrated that participants were unable to exploit across-trial spatial regularities regarding the salient distractors. These findings point to important boundary conditions for the modulation of visual attention by statistical regularities and they highlight the need to differentiate between different types of statistical regularities.

## Introduction

We are living in a world in which there is an overload of visual input. The ability to focus on the task at hand, ignoring irrelevant events, is critical in making behavior efficient. For instance, when crossing a busy road, pedestrians should focus on the traffic condition, while not being distracted by the salient billboards. Selective attention is the mechanism that enables us to effectively select relevant objects while simultaneously suppressing irrelevant information (Desimone & Duncan, 1995; Itti & Koch, 2001). It is traditionally assumed that attentional selection is influenced by the current goals of the observer and the physical saliency of the objects (Egeth & Yantis, 1997; Gaspelin & Luck, 2018; Theeuwes, 2010). In the past decade, however, it became clear that attention can also be biased toward objects that were unrelated to observers’ goal or were not physically salient (Awh et al., 2012). For example, an item that was attended on the previous trial (known as intertrial repetition priming; Maljkovic & Nakayama, 1996; Theeuwes & Van der Burg, 2013) or was previously associated with a monetary reward (e.g., Anderson et al., 2011; Failing & Theeuwes, 2014) would automatically capture observers’ attention. The search performance is facilitated when search scenes are repeatedly presented compared with new displays, known as contextual cueing, first examined by Chun and Jiang (1998). Therefore, “selection history” as originally was proposed by Awh et al. (2012) was considered a “third” category that could elicit attentional guidance, above and beyond goal-directed and stimulus-driven processes (see Anderson et al., 2021; Theeuwes, 2018; Theeuwes et al., 2022, for reviews).

Past experiences are accumulated so that the statistical regularities in the environment can be extracted by observers through a process called statistical learning (see Christiansen, 2019; Frost et al., 2019, for reviews). Since the seminal finding that 8-month-old infants can pick up repeated trisyllabic patterns from a continuous speech stream (Saffran et al., 1996), the amount of research on statistical learning has increasingly grown. Extending the findings for infants, a series of studies documented a remarkable sensitivity to the statistical patterns in sensory input for individuals of all ages (e.g., Batterink & Paller, 2019; Buiatti et al., 2009; Saffran et al., 1999), not only in the auditory but also in the visual modality, with different types of stimuli (e.g., Fiser & Aslin, 2002; Fiser et al., 2007; Turk-Browne et al., 2005; R. Yu & Zhao, 2018). Together, these findings document exhibiting the powerful learning abilities of humans in the extraction of temporally predictive relationships such as pairs, triplets, or longer sequences (see Frost et al., 2019, for a recent review).

A typical operationalization of visual statistical learning (VSL) concerning temporal regularities is that participants passively view a continuous stream of nonsense shapes (presented one shape at a time), which are structured into regular patterns—typically, pairs or triplets (e.g., Fiser & Aslin, 2002; Turk-Browne et al., 2005). Even though participants are typically not instructed to detect pairs or triplets, they are usually able to automatically extract the patterns in several minutes of exposure, expressing more familiarity with previously encountered patterns than with foils (i.e., the same shapes but reordered). In addition to this learning measure based on overt forced-choice judgements, implicit response times (RTs) measurements have also been used (Henin et al., 2021; Turk-Browne et al., 2005; Turk-Browne et al., 2010). For example, on each trial, participants were presented with a rapid and short continuous stream and were required to detect (as quickly as possible) the target shape which was defined ahead of each trial. Results showed that participants gave faster responses when the target was the second or third item of a pattern so that its occurrence could be predicted given the occurrence of the first item, demonstrating the implicit anticipation through VSL (see also Siegelman et al., 2018).

**Fig. 1:**
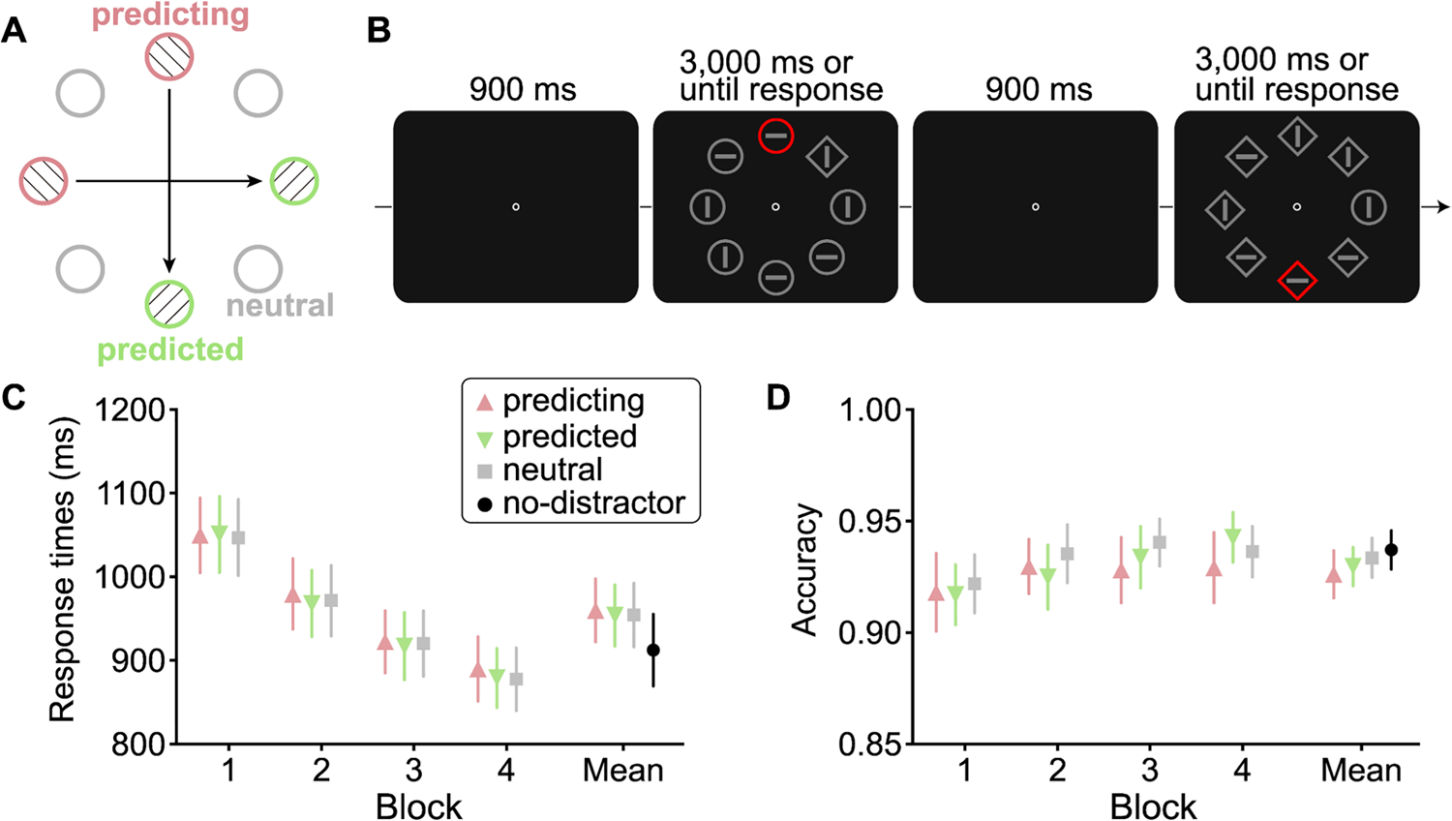
Stimuli and results in Experiment 1. **A** Illustration of two regularity pairs of two spatial locations each. A predicting trial (denoted with red circles for illustration purposes filled with left-oriented diagonal lines) predicted the location of the distractor on the subsequent trial (labelled as predicted; denoted with green circles filled with right-oriented diagonal lines). The locations of the distractor regularities were the same as locations of target regularities in Li and Theeuwes (2020). The neutral condition (denoted with unfilled grey circles) consisted of filler trials where the salient distractor appeared randomly at any other location. **B** Example of a stimulus display sequence with an across-trial regularity concerning colored distractor location. The colored distractor was always a red element presented among grey elements. **C** RTs as a function of distractor condition in each block. **D** Accuracy as a function of distractor condition in each block. Note that the no-distractor block was either before or after the four distractor-present blocks. The error bars denote 95% confidence intervals. (Color figure online)

In more recent years, VSL was also investigated in the context of visual search (see Theeuwes et al., 2022, for a recent review). It was shown that temporal predictive associations regarding spatial configurations and target locations in search displays can be implicitly learned and utilized to facilitate search (Boettcher et al., 2022; Li et al., 2022; Li & Theeuwes, 2020; Olson & Chun, 2001; Ono et al., 2005; Thomas et al., 2018; Toh et al., 2021). For instance, in the study by Li and Theeuwes (2020), participants were asked to search for a shape singleton target within a circular array of eight items (i.e., a diamond among seven circles or a circle among seven diamonds). The target was equally likely to appear at any of eight locations. The critical manipulation in this study was the introduction of two across-trial regularities of two target locations each (e.g., L_1_-> L_2_). This implied that if, on the current trial, the target was presented at a specific position L_1_ (predicting) in the display, on the following trial, the target was bound to appear at position L_2_ (predicted) with a 100% likelihood (see Fig. 1A). These trials were embedded amongst neutral trials in which the target appeared randomly at one of remaining four locations.

Both predicting and neutral were labelled unpredicted trials as the target location could not be predicted by the target location of previous trial. Interestingly, it was found that participants made faster and more accurate responses on predicted trials than unpredicted trials. Therefore, Li and Theeuwes (2020) concluded that the weights within the spatial priority map can be dynamically adapted on a trial-to-trial basis. It was reasoned that if a particular location was selected (the predicting location), the weight of the predicted (i.e., expected) location on the next trial was boosted, resulting in a faster selection of the predicted location. In a follow-up study using the *T*-among-*L*s task as an operationalization of slow inefficient serial search, Li et al. (2022) found that individuals did not express any RTs benefits on predicted trials. However, when observers were first exposed to the same regularities during parallel “pop-out” feature search (i.e., the target was colored differently from nontargets), across-trial VSL effects reoccurred, and the learned biases even persisted when search subsequently became inefficient and serial. These results revealed an important boundary condition of across-trial VSL and suggest that it might be easier for observers to learn the association between objects which are perceptually salient from the surroundings. This raises the question whether across-trial regularities can also modulate the attentional selection in visual search, if the regularities introduced are not task relevant but instead concern the locations of salient distractors.

Regularities regarding color singleton distractors are an interesting case because they do pop-out but, at the same time, are basically task irrelevant. Previous work has demonstrated that observers do adapt to simple distributional distractor regularities, where color singleton distractors are much more probable to occur at one particular location, as this high-probability distractor location becomes suppressed (Ferrante et al., 2018; Goschy et al., 2014; Wang & Theeuwes, 2018b). However, whether the attentional deprioritization of locations can also happen in a more dynamic fashion based on across-trial regularities is less clear. One may argue that it is unlikely that observers are able to learn across-trial spatial regularities of such salient distractors. Indeed, all VSL studies looking at across-trial location patterns mentioned above were about the location of the target, which is relevant and critically needs to be selected on each trial in order to be able to give the appropriate response. Especially when the target pops out from the search display, as soon as the display comes on, attention can be immediately shifted to the target location such that an association between target locations on consecutive two trials can be easily formed, without much, if any, interference. By contrast, a salient distractor is basically irrelevant to the task even though it is generally assumed that a salient distractor captures attention (Theeuwes, 1991, 1992). Still, compared with the target-target pairs, it is feasible that it is more difficult to form distractor-distractor associations across trials because in addition to attentional capture by the distractors, on each trial, there is the need to select the location of the target. Also, if attention is captured by the salient distractor, attention is typically disengaged extremely quickly (e.g., Born et al., 2011; Theeuwes et al., 2000), making it less likely that a strong memory trace for that location is formed.

However, two recent studies did show that observers can implicitly learn to suppress the location in advance that was likely to contain a distractor (i.e., proactive spatial suppression; see recent discussion in Gaspelin & Luck, 2021) on a trial-to-trial basis. In the study by Leber et al. (2016), a central arrow cue (e.g., pointing to the upper left) was presented at the beginning of each trial. It indicated the forthcoming target location with 70% validity, while it predicted the upcoming distractor location (e.g., bottom right) with 70% validity as well. Participants were only informed about the cue’s validity in predicting the target location. Surprisingly, participants did also pick up the relationship between the cue and the upcoming distractor location and used the learned regularities to flexibly reduce the distractor interference on a trial-to-trial basis. More related to across-trial VSL, in a recent study by Wang et al. (2021), using the additional singleton paradigm (Theeuwes, 1991, 1992), participants were asked to search for a specific shape (e.g., circle) throughout the experiment. Critically, for one group of participants, the salient distractor always moved to the adjacent location on the next trial in a clockwise or counterclockwise way while for another group of participants the across-trial order of the distractor locations was random. Based on the finding that the overall attentional capture of the regular group was significantly reduced compared with the random group, Wang et al. (2021) concluded that participants could learn across-trial distractor location regularities suggesting that participants proactively suppressed the anticipated (predicted) location of the distractor on the next trial. Yet it is clear that the Wang et al. (2021) study represents a special case in which the distractor is positioned throughout the experiment on the adjacent location on the next trial.

The current study tested whether participants are able to learn and use across-trial distractor location regularities just as they were able to learn across-trial target location regularities (Li et al., 2022; Li & Theeuwes, 2020). In Experiments 1 and 2, the same across-trial location pairs (within-subjects design) as Li and Theeuwes (2020) were used, except that it concerned regularities regarding the distractor instead of the target. In Experiments 3 and 4, we used a design similar to Wang et al. (2021), including separate distractor-absent blocks and distractor-present blocks to measure the possible attenuation of attentional capture. For the regular group, all possible distractor locations were structured into a long sequence, which was repeated throughout the experiment. For the random group, all trials were in a pseudorandom order. In Experiment 5, we sought to replicate the findings of Experiment 2 of Wang et al. (2021). If individuals could learn the spatial across-trial distractor regularities, RTs in the predicted condition would be faster than unpredicted condition (Experiments 1 and 2); the overall attentional capture in the regular group should be weaker than the random group (Experiments 3–5).

## Experiment 1

Experiment 1 was designed to examine whether observers can extract statistical regularities regarding the salient distractor locations across trials. Our prediction was straightforward: If participants can extract the across-trial distractor regularities, RTs for trials in which the distractor location is predicted by the previous trial should be faster than unpredicted trials (i.e., predicting and neutral conditions).

### Method

#### Participants

Our effect of interest was the main effect of the distractor regularity (predicted vs. unpredicted) in the two-way repeated-measures analysis of variance (RM-ANOVA), with block and distractor regularity as within-subject factors. An a priori power analysis was conducted using G*Power (Faul et al., 2007), with an alpha level of 0.05, 90% power, conservatively assumed correlation of *r* = 0.5 among repeated measures (see Brysbaert, 2019). Because no prior research regarding the effect of across-trial distractor regularities is available, we chose the recommended *f* = .2 (corresponding to η_p_^2^ = 0.04) as the smallest effect size of interest, which is the average effect size in psychological research (see Brysbaert, 2019). The suggested minimum sample size was 68 participants. We then recruited 68 participants (24 females, 43 males, and one other; *M*_age_ = 22.65 years, *SD*_age_ = 3.37) via Prolific (Palan & Schitter, 2018) and gave each participant a reward of £3.75. They all reported normal or corrected-to-normal visual acuity as well as normal color vision and gave informed consent before the experiment. The study was approved by the Ethics Committee of Department of Experimental and Applied Psychology of Vrije Universiteit Amsterdam.

#### Apparatus

The experiment was created in OpenSesame (Version 3.3.9b1) using OSWeb (Mathôt et al., 2012) and ran on a JATOS server (Lange et al., 2015). Participants were instructed to complete the task on their own computer or laptop in a quiet environment, after turning off all other possibly distracting electronic devices. The resolution specified in the experiment was 1,024 × 768 pixels (px). All stimuli were displayed on the “virtual monitor” in the center of the screen.

#### Procedure and design

Figure 1B illustrates the trial sequence on the distractor-present blocks. At the beginning of each trial, a white (RGB:255/255/255) fixation dot (16 × 16 px) was presented at the center of the screen on a black background (RGB: 0/0/0) and remained visible throughout the trial. After 900 ms, the search array which consisted of eight unfilled shapes (diamond: 100 × 100 px; circle: 94 × 94 px) positioned on an imaginary circle (radius: 224 px) was displayed for 3,000 ms or until the participant responded. Each shape was grey (RGB: 128/128/128) except the colored distractor was red (RGB: 255/0/0). Each shape contained either a horizontally or vertically oriented grey line (52 × 8 px). The task was to search for the unique shape (a diamond among circles, or vice versa, with equal likelihood) and report the orientation of the line segment inside of it (horizontal or vertical with equal probability). The search target was equally likely to appear at each of the eight locations and the red salient distractor was equally often presented at each of remaining seven locations. Participants were instructed to maintain fixation on the central dot throughout the experiment and to press the appointed response keys (“C” button for horizontal line, “M” button for vertical line) as fast and as accurately as possible. If participants did not respond correctly within 3000 ms, they would get a text display “Your response was wrong!” as well as an instruction screen repeating the correct key assignments for 800 ms. Feedback consisting of accuracy and mean RTs in the just completed block was given at the end of each block. Breaks between blocks were controlled by participants themselves.

Within each block, there were particular across-trial regularities regarding the distractor locations (see Fig. 1A). For all other trials within a block, distractors were positioned at randomly chosen locations. For half of the participants, as illustrated in Fig. 1A, if on the prior trial, a red distractor was presented at the top position of the display (predicting condition), it was always followed by a red distractor presented at the bottom position on the subsequent trial (predicted condition). For that same group of participants, there was another regularity: If the red distractor appeared at the leftmost position in the display (predicting condition), it was always followed by the red distractor appearing at the rightmost position (predicted condition). For the other half of participants, regularity pairs moved across trials in the opposite direction (bottom (B) → top (T), rightmost (R) → leftmost (L)). Note that these regularities only concerned the distractor location; its shape and the orientation of the line inside of it changed unpredictably from trial to trial. The regularity pairs were randomly intersected among the neutral trials in which the red distractor appeared at the other four locations, with the constraint that the same regularity pair could not repeat back-to-back (e.g., RLRL or BTBT was not allowed).

The experiment consisted of four blocks of distractor-present trials in which the regularities regarding the red distractor locations were built in, and a brief questionnaire containing three questions. Each block contained 112 trials, yielding 28 predicted trials, 28 predicting trials and 56 neutral trials. After participants had finished the distractor-present blocks, they were asked whether they were aware of any sequential relationship regarding the red distractor location such as one specific location was always followed by another. They were also asked to perform an eight-alternative forced-choice task (once for each regularity pair, twice in total) indicating which location the red distractor was most likely to appear after it was presented at the predicting location on the prior trial. All three questions were followed by a confidence judgment on a 5-point scale (1= *not certain at all*, 5 = *very certain*). In addition to the distractor-present blocks, participants also performed a single block of 112 distractor-absent trials either before or after the distractor-present trials (counterbalanced across participants). We presented distractor-present and distractor-absent trials in separate blocks to ensure optimal conditions for learning across-trial associations between distractor locations. Because in distractor-present blocks, a distractor was present on each trial, it was ensured that distractor–distractor associations could be formed between two consecutive trials, without the possibility that distractor-absent trials would weaken or prevent the formation of a possible association between two consecutive distractor-present trials. Before starting the formal experiment, a practice block containing 30 distractor-absent trials and 30 distractor-present trials in the randomized order was repeated until the participant reached the criteria of accuracy >85% as well as mean RTs <1,400 ms.

### Results

#### Analysis

RTs were limited to trials with correct responses (93.13%). For the remaining trials within each block RTs of each participant were submitted to a non-recursive trimming procedure (Vanselst & Jolicoeur, 1994) that uses cell size to determine a criterion number of standard deviations (*SD*s) from the mean beyond which an observation is considered as an outlier (2.84%). Then, trials with RTs faster than 200 ms (0.05%) were also excluded from analysis. In distractor-present trials, if the distractor happened to be presented at the same location twice, these trials were removed to exclude effects of repetition location priming (5.29%). Mean RTs and accuracies were first submitted into a one-way (distractor condition: predicting, predicted, neutral and no-distractor) RM-ANOVA, and the data on distractor-present blocks were then submitted into a two-way RM-ANOVA (Block × Regularity). Greenhouse–Geisser corrected *p* values (*p*_c_) were used in case of sphericity assumption violations. In addition, whenever a comparison using traditional null hypothesis testing was nonsignificant, we also quantified the Bayes factor (BF) using Bayesian hypothesis testing in JASP (Wagenmakers et al., 2018) to evaluate the strength of the evidence for the alternative hypothesis (H1) over the null hypothesis (H0).

#### Attentional capture

A one-way RM-ANOVA on RTs with the distractor condition as the factor revealed a significant main effect, *F*(3, 201) = 7.43, *p*_c_ < .001, η_p_^2^ = 0.10. Post hoc tests indicated that relative to RTs in the no-distractor condition (913 ms), all three distractor-present conditions showed significantly longer RTs (predicted: 960 ms), *t*(67) = 3.09, *p =* .003, Cohen’s *d* = 0.37; (predicting: 954 ms), *t*(67) = 2.62, *p =* .011, Cohen’s *d* = 0.32; (neutral: 955 ms), *t*(67) = 2.73, *p =* .008, Cohen’s *d* = 0.33. The overall attentional capture (distractor-present – distractor-absent) in RTs was on average 43 ms. A one-way RM-ANOVA on accuracy also showed a significant main effect of distractor condition, *F*(3, 201) = 2.74, *p* = .044, η_p_^2^ = 0.04. Post hoc tests further revealed that the accuracy of no-distractor condition (93.71%) was significantly higher than predicting condition (92.62%), *t*(67) = 2.69, *p* = .009, Cohen’s *d* = 0.33.

#### Learning effect

Figure 1C and D show the RTs and accuracies as a function of regularity for every block. The two-way RM-ANOVA with block and regularity as factors only revealed a significant main effect of block, *F*(3, 201) = 80.64, *p*_c_ < .001, η_p_^2^ = 0.55. Neither the main effect of regularity, *F*(2, 134) = 1.50, *p* = .23, η_p_^2^ = 0.02, BF_01_ = 49.26 (strong evidence for the absence of any difference) nor the Block × Regularity interaction, *F*(6, 402) = 0.42, *p* = .87, η_p_^2^ = 0.01, BF_01_ = 755.38 (strong evidence for the absence of the interaction effect) reached significance. The same analysis on accuracy showed a significant main effect of block, *F*(3, 201) = 5.57, *p*_c_ = .002, η_p_^2^ = 0.08. The main effect of regularity was unreliable, *F*(2, 134) = 1.61, *p* = .20, η_p_^2^ = 0.02, BF_01_ = 14.44. The Block × Regularity interaction did also not reach significance, *F*(6, 402) = 0.69, *p* = .66, η_p_^2^ = 0.01, BF_01_ =166.64.

As target and distractor shapes randomly swapped from trial to trial, it is possible to examine the contribution of feature-based suppression. To that end we conducted a two-way RM-ANOVA with shape repetition (repeat vs. switch) and regularity (predicted, predicting and neutral) as factors on RTs. The results indicated a significant effect of shape repetition with faster response times for repeat trials (909 ms) than for switch trials (1,005 ms), *F*(1, 67) = 238.33, *p* < .001, η_p_^2^ = 0.78. Critically however, there was no main effect of regularity, *F*(2, 134) = 1.51, *p* = .22, η_p_^2^ = 0.02, BF_01_ = 25.97, nor an interaction between shape repetition and regularity, *F*(2, 134) = 2.05, *p* = .13, η_p_^2^ = 0.03, BF_01_ = 4.87 (moderate evidence for the absence of the interaction), suggesting that the feature repetition did not modulate across-trial learning of distractor locations.

#### Awareness of the regularities

Thirteen out of 68 participants reported to have been aware of the across-trial association of the red distractor locations during the experiment, with a mean confidence score (CS) of 3.69*.* Yet only one of them correctly chose both predicted locations (CS: 2.5), while others indicated incorrect locations (CS: 2.54). The remaining 55 participants reported to be unaware of the across-trial distractor location regularities (CS: 3.44). The mean CS regarding locations they chose was 1.56.

### Discussion

In this experiment we tested whether observers can make use of spatial across-trial distractor regularities to facilitate visual search. We observed no RTs benefit of predictable trials compared with unpredictable trials, indicating that participants did not learn the distractor regularities. It is possible that participants did not learn the spatial across-trial regularities because the distractor was not salient enough to capture (enough) attention on each trial. Note the overall capture effect in the present experiment was only about 43 ms. There is evidence showing that when participants have specific target templates, the repeated salient distractor only captures attention in the beginning of the experiment (Vatterott & Vecera, 2012). Participants knew that the target was always grey (and therefore never red) making it likely that not on each trial attention was captured by the distractor. If attention is not captured by the distractor on those critical trials in which the regularities occurred, then might be difficult (maybe even impossible) to learn these across-trial regularities. In the next experiment we hence ensured that capture was much stronger, providing optimal opportunity to learn the across-trial regularities regarding the distractor.

## Experiment 2

To make attentional capture by the color distractor stronger, in Experiment 2 the colors of target and distractor swapped randomly from trial to trial which is known to increase attentional capture (Pinto et al., 2005).

### Method

**Fig. 2:**
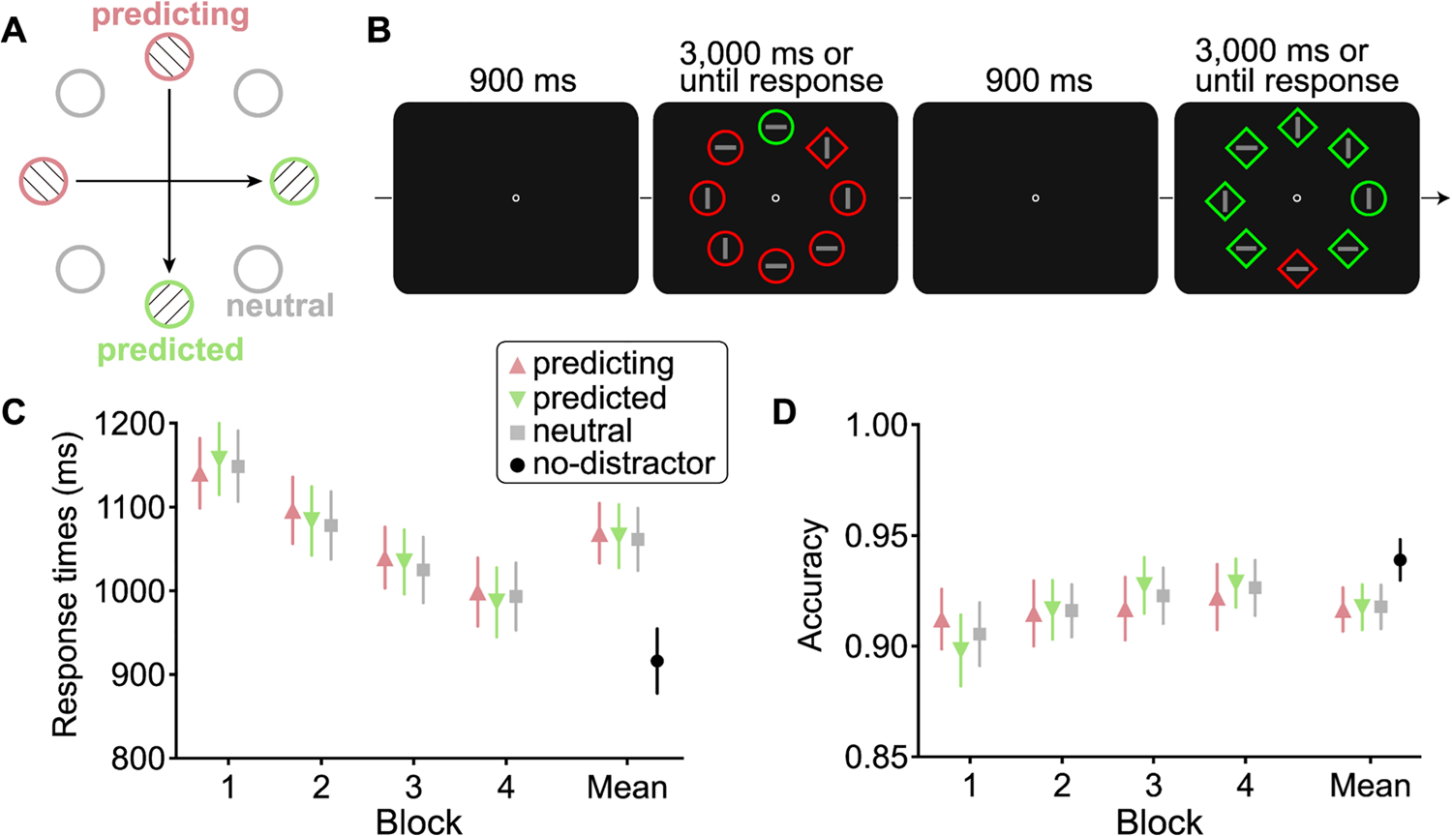
Stimuli and results of Experiment 2. **A** Illustration of two regularity pairs as Experiment 1. **B** Example of a stimulus display sequence with an across-trial regularity concerning colored distractor location. The colored distractor was randomly red among green items or green among red items. **C** RTs as a function of distractor condition in each block. **D** Accuracy as a function of distractor condition in each block. Note that the no-distractor block was either before or after the four distractor-present blocks. The error bars indicate 95% confidence intervals. (Color figure online)

The method was identical to that of Experiment 1, with the following changes. First, a new set of 68 participants (21 females, 44 males, and three others, *M*_age_ = 22.82 years, *SD*_age_ = 4.55) were recruited via Prolific, and each was rewarded £4.5 for completing the whole experiment. All participants reported normal color vision as well as normal or corrected-to-normal visual acuity. Second, as illustrated in Fig. 2B, the colors of target and distractor swapped randomly from trial to trial. In other words, the colored distractor was equally likely to be red among green and green among red items.

### Results

#### Analysis

RTs were limited to correct trials (92.28%). With the same trimming procedure as Experiment 1, trials with slow RT outliers (2.60%) and with RTs faster than 200 ms (0.01%) were excluded from analysis. Repetition distractor location priming trials (5.35%) were also rejected. The same analyses as in Experiment 1 were conducted on RTs and accuracies.

#### Attentional capture

A one-way RM-ANOVA on RTs revealed a significant main effect of distractor condition, *F*(3, 201) = 81.52, *p*_c_ < .001, η_p_^2^ = 0.55. Post hoc tests indicated that relative to RTs in the no-distractor condition (916 ms), all three distractor-present conditions showed longer RTs (predicted: 1,069 ms), *t*(67) = 9.66, *p* < .001, Cohen’s *d* = 1.17; (predicting: 1,065 ms), *t*(67) = 9.85, *p* < .001, Cohen’s *d* = 1.19; (neutral: 1,062 ms), *t*(67) = 8.84, *p* < .001, Cohen’s *d* = 1.07. The attentional capture in RTs was on average 149 ms. A one-way RM-ANOVA on accuracies also showed a significant main effect of distractor condition, *F*(3, 201) = 10.35, *p* < .001, η_p_^2^ = 0.13. Post hoc tests showed that the accuracy of no-distractor condition (93.91%) was significantly higher than the predicted (91.66%), *t*(67) = 4.77, *p* < .001, Cohen’s *d* = 0.58; predicting (91.77%), *t*(67) = 3.75, *p* < .001, Cohen’s *d* = 0.45; and neutral (91.79%), *t*(67) = 4.99, *p* < .001, Cohen’s *d* = 0.59, condition. All these results indicate strong attentional capture by the color singleton distractor.

#### Learning effect

Mean RTs and accuracies as a function of regularity for each block are shown in Fig. 2C and D, respectively. The two-way RM-ANOVA with block and regularity as factors on RTs only revealed the significant main effect of block, *F*(3, 201) = 78.48, *p*_c_ < .001, η_p_^2^ = 0.53. Neither the main effect of regularity, *F*(2, 134) = 1.17, *p* = .31, η_p_^2^ = 0.02, BF_01_ = 48.16 (strong evidence for the absence of any difference between conditions), nor the Block × Regularity interaction, *F*(6, 402) = 1.54, *p* = .17, η_p_^2^ = 0.02, BF_01_ = 121.24 (strong evidence for the absence of the interaction effect), reached significance. The same analysis was conducted on accuracy. Results only showed a significant main effect of block, *F*(3, 201) = 9.72, *p*_c_ < .001, η_p_^2^ = 0.13. The main effect of regularity was unreliable, *F*(2, 134) = 0.04, *p* = .96, η_p_^2^ = 0.00, BF_01_ = 67.73. The Block × Regularity interaction did also not reach significance, *F*(6, 402) = 1.00, *p* = .43, η_p_^2^ = 0.01, BF_01_ = 82.85.

Similar to Experiment 1, we examined the contribution of feature-based suppression. We conducted a three-way RM-ANOVA with shape repetition (repeat vs. switch), color repetition (repeat vs. switch) and regularity (predicted, predicting and neutral) as factors on RTs. Again, there was a significant main effect of shape repetition (repeat vs. switch: 1,021 vs. 1,110 ms), *F*(1, 67) = 271.76, *p* < .001, η_p_^2^ = 0.80. The main effect of color repetition was also significant, *F*(1, 67) = 20.85, *p* < .001, η_p_^2^ = 0.24, with faster RTs for repeat trials (1053 ms) than for switch trials (1,078 ms). However, neither color repetition × regularity interaction, *F*(2, 134) = 1.18, *p* = .31, η_p_^2^ = 0.02, BF_01_ = 11.97, nor Shape Repetition × Regularity interaction, *F*(2, 134) = 2.86, *p* = .06, η_p_^2^ = 0.04, BF_01_ = 4.05, was significant. The three-way interaction also did not reach significance, *F*(2, 134) = 1.04, *p* = .35, η_p_^2^ = 0.02, BF_01_ = 9.56. These moderate to strong evidence for the absence of the interaction suggests again no significant contribution of feature repetition to across-trial learning of distractor locations.

#### Awareness of the regularities

Twelve out of 68 participants reported to have been aware of the across-trial association of the red distractor location during the experiment (CS: 3.75). Yet only one of them correctly chose both of the predicted locations (CS: 3), while others indicated wrong locations (CS: 2.77). The remaining 56 participants reported to be unaware of the across-trial distractor location regularities (CS: 3.52). The mean CS regarding locations they chose was 1.98.

### Discussion

By swapping the target and distractor colors randomly from trial to trial, the attentional capture (in RTs) was about 3.5 times stronger than the capture effect in Experiment 1. Even though the salient color distractor now strongly captured attention, there was still no difference between predicted and unpredicted conditions. The results suggest that it is difficult to learn and exploit across-trial regularities regarding the distractor location, even when the distractor is highly salient. The current results are inconsistent with the findings of Wang et al. (2021), who demonstrated that if possible distractor locations are bound to a repeating and consistent sequence, attentional capture was reduced. This implies that under specific circumstances statistical learning of across-trial distractor contingencies are possible.

Even though the study of Wang et al. (2021) showed across-trial distractor learning, one aspect of this study may be considered to be problematic: In the regular group the across-trial distractor distance was always one (i.e., average distance of one), while in the random group it was randomized to be 0/1/2/3/4 (i.e., average distance of around two). It is known that when the salient distractor appears at the same location as in the previous trial, participants can respond faster because the previous distractor location has been suppressed (e.g., Gotts et al., 2012) and such suppression can spread to surrounding locations. The question is then whether Wang et al. (2021) represents true statistical learning of the sequence, or whether the results can be explained by across-trial spreading of suppression to nearby locations. Therefore, in the following experiment, we presented the salient distractor in a regular versus random across-trial sequence, while controlling for the across-trial distractor distances in both groups.

## Experiment 3

The previous experiments did not show any evidence for learning of paired across-trial regularities regarding distractor locations. Instead of just using pairs, we aimed to investigate whether it is possible for participants to learn distractor location regularities consisting of longer sequences. In this experiment the salient distractor location was presented in a particular fixed order for one group of participants (the regular group), while it was presented in a pseudorandom order for another group (the random group). If participants in the regular group would pick up the across-trial sequence of salient distractor locations, it is expected that the overall attentional capture effect of the regular group would be reduced compared with that of the random group.

### Method

The method was identical to Experiment 2, with the following changes. First, our effect of interest was the Group × Distractor Presence interaction effect of a three-way ANOVA, with block (1–4) and distractor presence (present vs. absent) as the within-subjects factors, group (regular vs. random) as the between-subjects factor. An a-priori power analysis was conducted using G*Power (Faul et al., 2007), with α = 0.05, power = 0.9, effect size *f* = 0.2 (corresponding to η_p_^2^ = .04), correlation among repeated measures *r* = 0.5, the minimum total sample size was calculated to be 66 participants. A new set of 80 participants (30 females and 50 males, *M*_age_ = 23.21 years, *SD*_age_ = 5.39) were recruited via Prolific, randomly assigned to one of two groups (40 participants per group).

**Fig. 3:**
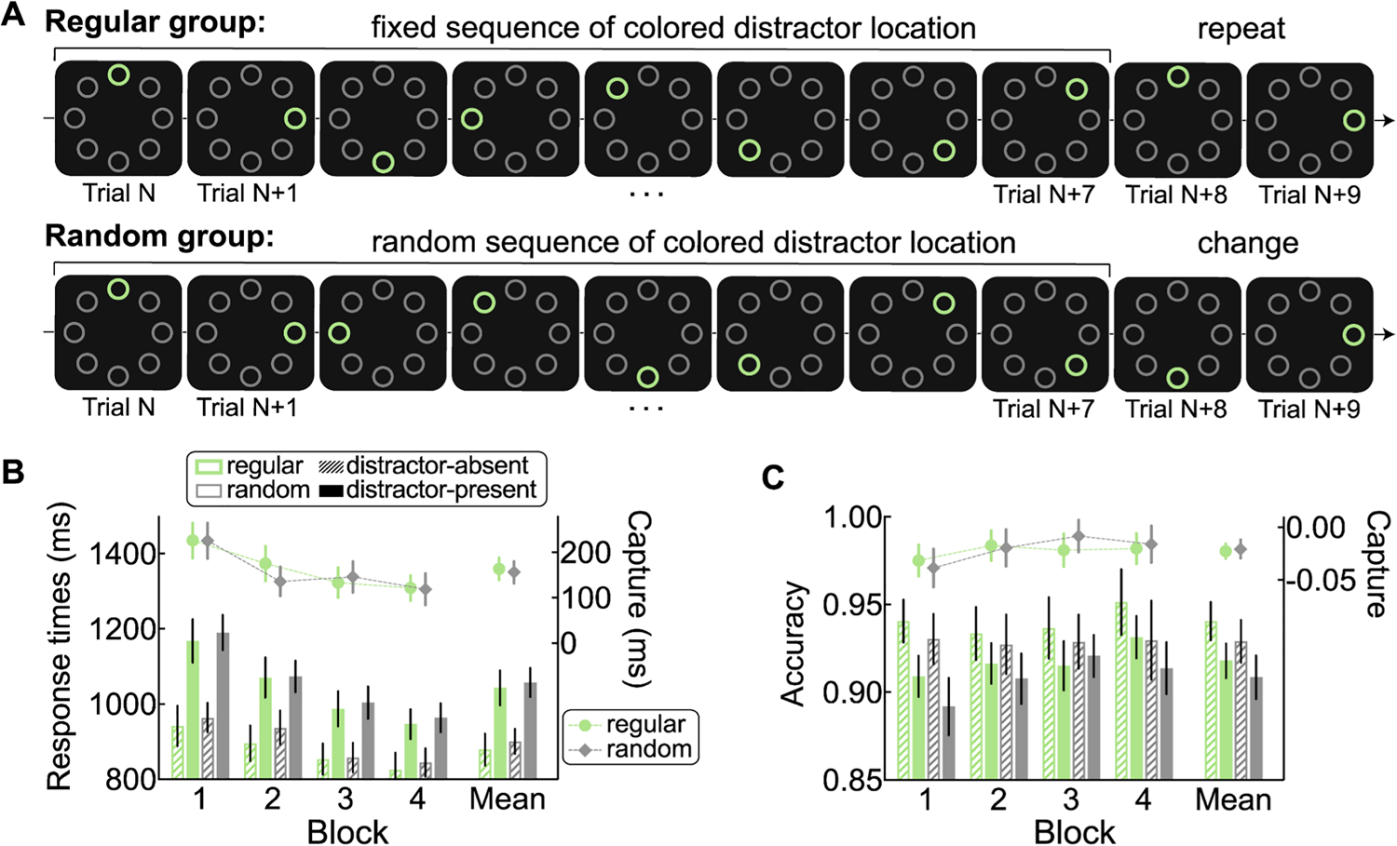
Build-in regularity pattern and results in Experiment 3. **A** Example of the sequence regarding the colored distractor location (denoted by the green circle, only for the illustration purpose) in the distractor-present blocks for the regular group and random group. In the real experiment, the colors (red and green) and shapes (diamond and circle) of target and distractor swapped randomly from trial to trial. **B** RTs (left *y*-axis) as a function of distractor condition (distractor-absent and distractor-present) and corresponding capture effect (distractor-present – distractor-absent, right *y*-axis) in each block for the regular group and random group. **C** Accuracies (left *y*-axis) as a function of distractor condition and corresponding capture effect (distractor-present – distractor-absent, right *y*-axis) in each block for the regular group and random group. The error bars indicate 95% confidence intervals. (Color figure online)

Second, the distractor locations were presented sequentially for the regular group and in a pseudorandom order for the random group. Specifically, for the regular group, the distractor location was displayed according to a fixed sequence which was repeated 14 times within one distractor-present block. The average across-trial distance of distractor locations was 1.75, with a *SD* of 0.4629 (e.g., 1-3-5-7—8-6-4-2, as illustrated in Fig. 3A). We reasoned that such sequential regularities should be learned easier than paired regularities as used in Experiments 1 and 2. Note that in these previous experiments, the paired regularities were embedded among random trials. Hence in most cases the distractor location was picked randomly and only in 25% of trials it was predictable. In comparison, in the current experiment, in the sequential condition, all the trials were predictable and the transitions between trials were quite similar (i.e., the singleton distractor moved two locations towards the same direction on consecutive several trials). For each participant of the regular group, the fixed sequence was randomly selected from 16 possible sequences (eight starting locations × clockwise/counter-clockwise starting rotation) and kept consistent throughout the whole experiment. For each participant of the random group, 14 different sequences were used per block. Each sequence consisted of eight locations in random order, with two constraints: (1) no repetition of the salient distractor location when crossing sequences; (2) the average across-trial distance of the salient distractor location within the whole block was 2.

Participants first finished the practice block (same practice as in Experiment 2), and then completed four experimental blocks. Each experimental block contained a mini-block of 112 distractor-present trials and another mini-block of 32 distractor-absent trials. The order of mini-block types (distractor-absent and distractor-present) was counterbalanced across participants and remained the same in all four blocks. After each mini-block, feedback on the average RTs and accuracy was given. In the end of the experiment, participants of the regular group were asked to recall whether they were aware of the sequential relationship regarding the salient distractor locations. They were also asked to answer four eight-alternative forced-choice questions to indicate at which location the salient distractor was most likely to appear after it was presented at the predicting location on the prior trial (i.e., the 1st, 3rd, 5th, 7th or 2nd, 4th, 6th, 8th location of the sequence). The order of the four eight-alternative forced-choice questions was counterbalanced among participants. All questions were followed by a confidence judgment on a 5-point scale (1= *not certain at all*, 5 = *very certain*).

### Results

#### Analysis

RTs were limited to correct trials (91.81%). For the remaining trials within each mini-block of each participant, trials with slow outliers (2.72%) using the same trimming procedure as Experiment 1 and with RTs faster than 200 ms (0.02%) were excluded from analysis.

#### Attentional capture

Figure 3B and C show the RTs and accuracies as a function of distractor presence and corresponding capture effect (the difference between distractor-present and distractor-absent conditions) in separate blocks for the regular group and random group. A three-way mixed ANOA with block (1–4) and distractor presence (absent vs. present) as the within-subjects factor, and group as the between-subjects factor was conducted on mean RTs. The main effect of block was significant, *F*(3, 234) = 138.86, *p*_c_ < .001, η_p_^2^ = 0.64, but not the Group × Block interaction, *F*(3, 234) = .19, *p* = .90, η_p_^2^ = 0.00, BF_01_ = 43.73. The main effect of distractor presence was also significant, *F*(1, 78) = 366.24, *p*_c_ < .001, η_p_^2^ = 0.82, but not the Group × Distractor Presence interaction, *F*(1, 78) = 0.21, *p* = .65, η_p_^2^ = 0.00, BF_01_ = 6.94. The Block × Distractor Presence interaction was significant, *F*(3, 234) = 20.34, *p* < .001, η_p_^2^ = 0.21, but not the Group × Block × Distractor Presence interaction, *F*(3, 324) = 1.20, *p* = .31, η_p_^2^ = 0.02, BF_01_ = 11.88. We also did independent-samples *t* tests, for each block, comparing RTs in each distractor presence condition between the two groups (all *p*s > .19). Similarly, we also tested, for each block, for a group difference in the capture effect of RTs (all *p*s > .11). These results suggest that there was no difference between two groups, whether in raw RTs or in the capture effect.

The same analyses conducted on accuracies showed similar results as RTs and we only report here important results. First, we did not observe a significant Group × Distractor Presence interaction, *F*(1, 78) = 0.13, *p* = .72, η_p_^2^ = 0.00, BF_01_ = 7.54, or a significant Group × Block × Distractor Presence interaction, *F*(3, 324) = 0.63, *p* = .60, η_p_^2^ = 0.01, BF_01_ = 16.94. Second, there was no difference between two groups in capture effect of accuracies on each block (all *p*s > .21).

#### Awareness of the regularities

Seven out of 40 participants reported to have been aware of the across-trial association of the red distractor location during the experiment (CS: 3.71). However, all of them chose three or four incorrect predicted locations (CS: 3.00). The remaining 33 participants reported to be unaware of the across-trial distractor location regularities (CS: 3.73). The mean CS regarding locations they chose was 2.00.

### Discussion

In Experiment 3, we utilized a consistently repeating sequence of distractor locations for the regular group while controlling the across-trial distance within the whole block for the random group. We did not observe any difference between the two groups in raw RTs/accuracies or the capture effect of RTs/accuracies. Despite the use of a fixed sequence of distractor locations, which we hypothesized this would be much easier to detect than the pair regularity in the previous two experiments, the regular sequence was still relatively complex as it involved two subsequences with different rotation directions (clockwise and counterclockwise). In the next experiment, we used an easier pattern which only involved one rotation direction. Unlike Wang et al. (2021), on the next trial, the distractor location always moved two items instead of only one.

## Experiment 4

In Experiment 4, we used an easier sequence to investigate the possibility that when the across-trial regularities regarding the distractor location are relatively simple they can be learned and used to reduce the interference caused by distractors. For the regular group, the salient distractor location always moved either clockwise or counterclockwise, in steps of two items.

### Method

**Fig. 4:**
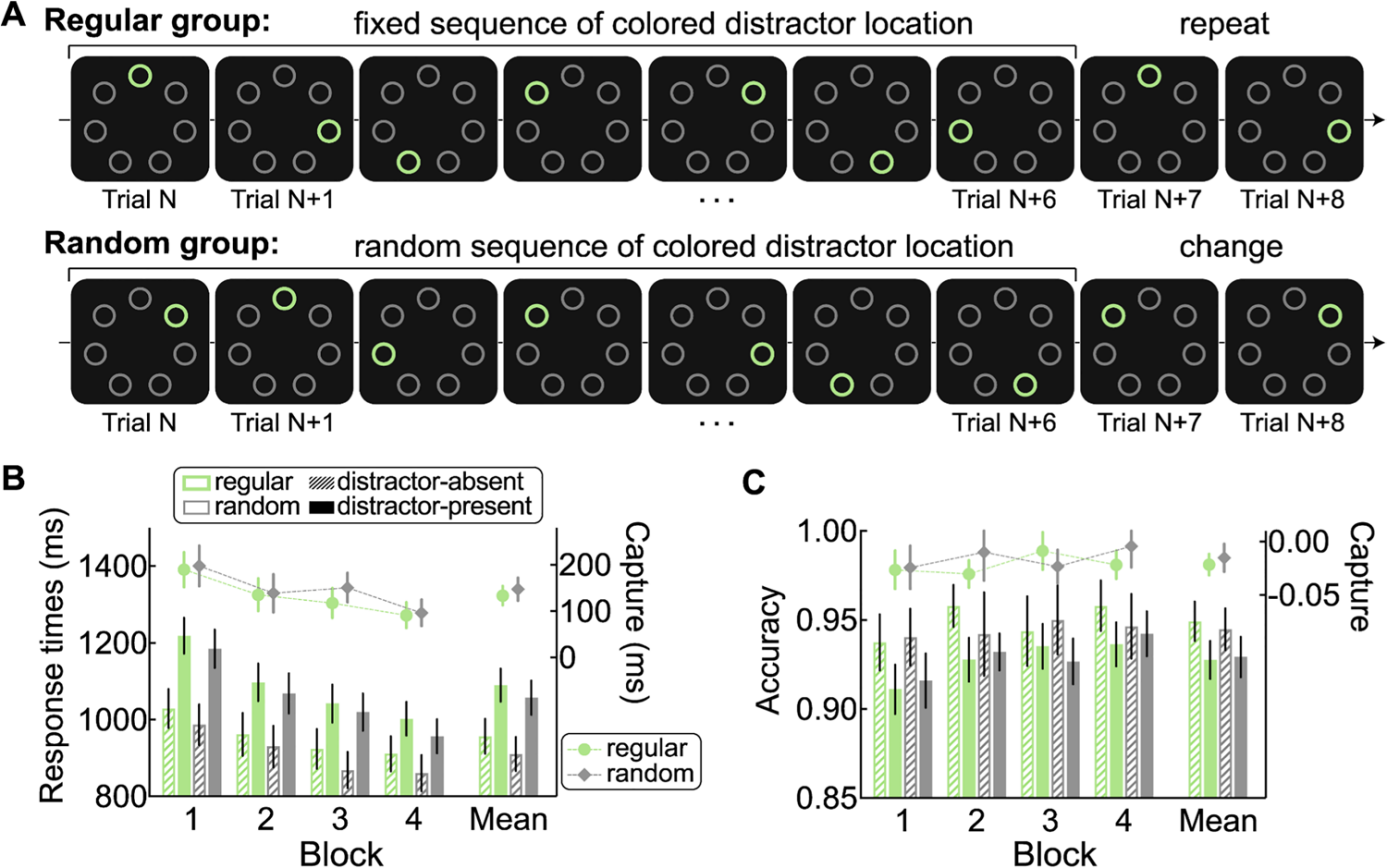
Build-in regularity pattern and results in Experiment 4. **A** Example of the sequence regarding the colored distractor location (denoted by the green circle) for the regular group and random group. In the real experiment, the colors (red and green) and shapes (diamond and circle) of target and distractor swapped randomly from trial to trial. **B** RTs (left *y*-axis) as a function of distractor condition (distractor-absent and distractor-present) and corresponding capture effect (distractor-present – distractor-absent, right *y*-axis) in each block for the regular group and random group. **C** Accuracies (left *y*-axis) as a function of distractor condition and corresponding capture effect (right *y*-axis) in each block for the regular group and random group. The error bars indicate 95% confidence intervals. (Color figure online)

The method was identical to Experiment 3, with the following changes. First, a new set of 80 participants (35 females, 42 males, and three others, *M*_age_ = 22.64 years, *SD*_age_ = 3.30) were recruited via Prolific (40 participants per group). Second, as illustrated in Fig. 4A, a circular array of seven items was used because learning a sequence that is repeated across seven items should be easier than across nine items. For the regular group, the fixed sequence (e.g., 1-3-5-7-2-4-6; moving rotation of clockwise or counterclockwise was counterbalanced across participants, starting location was randomly selected for each participant) was repeated 14 times in one distractor-present mini-block, always with an across-trial distractor distance of two. To increase the potential RT benefits of using across-trial distractor location regularities, we set the restriction that the target could not appear at the intermediate location between the distractor locations on two consecutive trials. To make sure a difference between two groups could only due the presence of the across-trial regularity, rather than the trials themselves, the same target-distractor layout restriction was also implemented for the random group. For each participant of random group, each distractor-present mini-block contained 14 different sequences (locations 1–7 in random order) with the same constraints as in Experiment 3. Third, each block contained one mini-block of 98 distractor-present trials and one mini-block of 28 distractor-absent trials. Participants of the regular group were asked to answer four seven-alternative forced-choice questions to indicate at which location the salient distractor was most likely to appear after it was presented at the predicting location on the prior trial (i.e., 1st, 3rd, 5th, 7th or 2nd, 4th, 6th, 1st location of the sequence, with the order of the questions counterbalanced across participants).

### Results

#### Analysis

RTs were limited to correct trials (93.26%). For the remaining trials within each mini-block of each participant, using the same trimming procedure as Experiment 3, trials with slow outliers (2.69%) and with RTs faster than 200 ms (0.01%) were excluded from analysis.

#### Attentional capture

Figure 4B and C show the RTs and accuracies as a function of distractor presence and the calculated capture effect (distractor-present – distractor-absent) in each block for the regular group and random group. Mean RTs were submitted into a three-way mixed ANOVA with block and distractor presence as within-subjects factors and with group as between-subjects factor. The main effect of block was significant, *F*(3, 234) = 123.04, *p*_c_ < .001, η_p_^2^ = 0.61, but not the Group × Block interaction, *F*(3, 234) = 0.28, *p*_c_ = .81, η_p_^2^ = 0.00, BF_01_ = 57.59. The main effect of distractor presence was significant, *F*(1, 78) = 315.89, *p*_c_ < .001, η_p_^2^ = 0.80, but not the Group × Distractor Presence interaction, *F*(1, 78) = 0.61, *p* = .44, η_p_^2^ = 0.00, BF_01_ = 15.87. The Block × Distractor Presence interaction was significant as well, *F*(3, 234) = 14.33, *p* < .001, η_p_^2^ = 0.16, but not the Group × Block × Distractor Presence interaction, *F*(3, 324) = 0.42, *p* = .74, η_p_^2^ = 0.01, BF_01_ = 22.25. We did independent-samples *t* tests, for each block separately, on RTs of each distractor presence condition, but for none of the blocks a significant difference between groups was observed (all *p*s > .11). The capture effect of RTs in each block was also submitted into independent-samples *t* tests but, again, no difference between groups was found (all *p*s > .14).

Analyses on accuracies showed the similar results as RTs. Only important results regarding the difference between two groups were reported. The three-way ANOVA did not show a significant Group × Distractor Presence interaction, *F*(1, 78) = 0.62, *p* = .43, η_p_^2^ = 0.01, BF_01_ = 4.66, or a significant Group × Block × Distractor Presence interaction, *F*(3, 324) = 1.96, *p* = .12, η_p_^2^ = 0.02, BF_01_ = 5. Again, independent-samples *t* tests on the capture effect in accuracies in each block did not reveal any difference between two groups (all *p*s > .14).

#### Across-trial distance of distractor location

In this additional control analysis, for distractor-present trials in the random group, we restricted the across-trial distractor distance to two. A mixed ANOVA with the within-subjects factor distractor condition (distractor-absent vs. distractor-present) and the between-subjects factor group (regular vs. random) was conducted on mean RTs. Neither the main effect of group, *F*(1, 78) = 1.50, *p* = .22, η_p_^2^ = 0.02, BF_01_ = 1.91, nor interaction effect, *F*(1, 78) = 1.52, *p* = .22, η_p_^2^ = 0.02, BF_01_ = 2.58, was significant.

#### Awareness of the regularities

Eight out of 40 participants reported to have been aware of the across-trial association of the salient distractor location during the experiment (CS: 3.13). Two of them correctly chose three or four predicted locations (CS: 3), six of them correctly chose predicted locations less than three times (i.e., 0/1/2, CS: 2.92). The remaining 32 participants reported to be unaware of the across-trial distractor location regularities (CS: 3.44). The mean CS regarding locations they chose was 2.19.

### Discussion

In this experiment, we used a much easier regular pattern (i.e., the distractor location moved in steps of two locations, in a clockwise or counterclockwise way). Despite the use of a simple distractor location sequence, participants from the regular group still failed to express benefits in attentional capture compared with the random group. There were only two participants really “aware” of the regularity in the sense that they could also correctly report most of the regularity. Whereas the lack of evidence for across-trial learning of distractor location regularities in visual search in the current experiment is consistent with the three previous experiments, it is inconsistent with the findings of Wang et al. (2021). There are two differences between the current study and Wang et al. (2021). First, in Wang et al. (2021) the regular sequence implied that the distractor always moved to the adjacent location on the next trial, whereas distractor locations were random for the random group. This means that the average across-trial distance of distractor locations was not controlled (and indeed cannot be controlled) between regular group and random group. In the current experiment the regular sequence had steps of two locations, allowing us to equate the across-trial distractor distance between two groups. Second, throughout the experiment in Wang et al. (2021), the target and distractor had distinct shapes and colors (e.g., for a given participant the target was always a circle while the singleton distractor was a diamond; the target was grey while the salient distractor was either red or green). By contrast, in the present study, the shapes and colors of the target and distractor swapped unpredictably from trial to trial. In other words, the distractor feature in our study was more variable (lack of consistency), possibly making the spatial distractor regularity more difficult to learn. Importantly, a very recent study by H. Yu et al. (2022) using the same paradigm as our Experiments 3 and 4 also failed to replicate Wang et al. (2021). In their study, the distractor location was shifted to an adjacent location on 80% of trials (frequent) in a clockwise or counterclockwise way but shifted to the adjacent location in the opposite direction on 10% of trials (infrequent). On remaining 10% of trials, the distractor was presented at one of six nonadjacent locations (random). The results revealed no differences among three distractor conditions, basically providing a failure to replicate Wang et al. (2021).

Even though one may argue that the difference between current study and Wang et al. (2021) is rather small, it is unclear whether across-trial distractor location learning is possible yet limited to a few easy-to-learn regularities, for example the one that was tested by Wang et al. (2021). Therefore, we ran a new experiment that closely mimicked the paradigm of Experiment 2 of Wang et al. (2021).

## Experiment 5

This final experiment was closely modelled after Experiment 2 of Wang et al. (2021) to determine whether we could replicate their results. For the regular group, the salient distractor always moved to the adjacent location on the next trial in a clockwise or counterclockwise way while for the random group the salient distractor location was completely random.

### Method

A new set of 80 participants (26 females, 49 males, and five others, *M*_age_ = 24.51 years, *SD*_age_ = 3.42) were recruited via Prolific (40 participants per group). The method was identical to our Experiment 3, with the following changes to mimic the paradigm of Experiment 2 of Wang et al. (2021). First, the target shape was always fixed for a given participant (e.g., a diamond among circles) and was counterbalanced across participants; all shapes were grey except the salient distractor was either red (RGB: 255/0/0) or green (RGB: 0/255/0) with equal probability. Second, for the regular group, the colored distractor always moved to the adjacent location in a clockwise or counterclockwise direction, while for the random group the distractor location was completely random without any constraint. Third, each block contained one mini-block of 80 distractor-present trials and one mini-block of 40 distractor-absent trials. Lastly, all participants completed only one practice block consisting of 10 distractor-absent trials and 10 distractor-present trials.

### Results

#### Analysis

RTs were limited to correct trials (93.17%). For the remaining trials within each mini-block of each participant, using the same trimming procedure as Experiment 3, trials with slow outliers (2.65%) and with RTs faster than 200 ms (0.02%) were excluded from analysis. For the random group, if the salient distractor appeared at the same location twice, these trials were removed to exclude effects of repetition location priming (6.72%).

#### Attentional capture

**Fig. 5:**
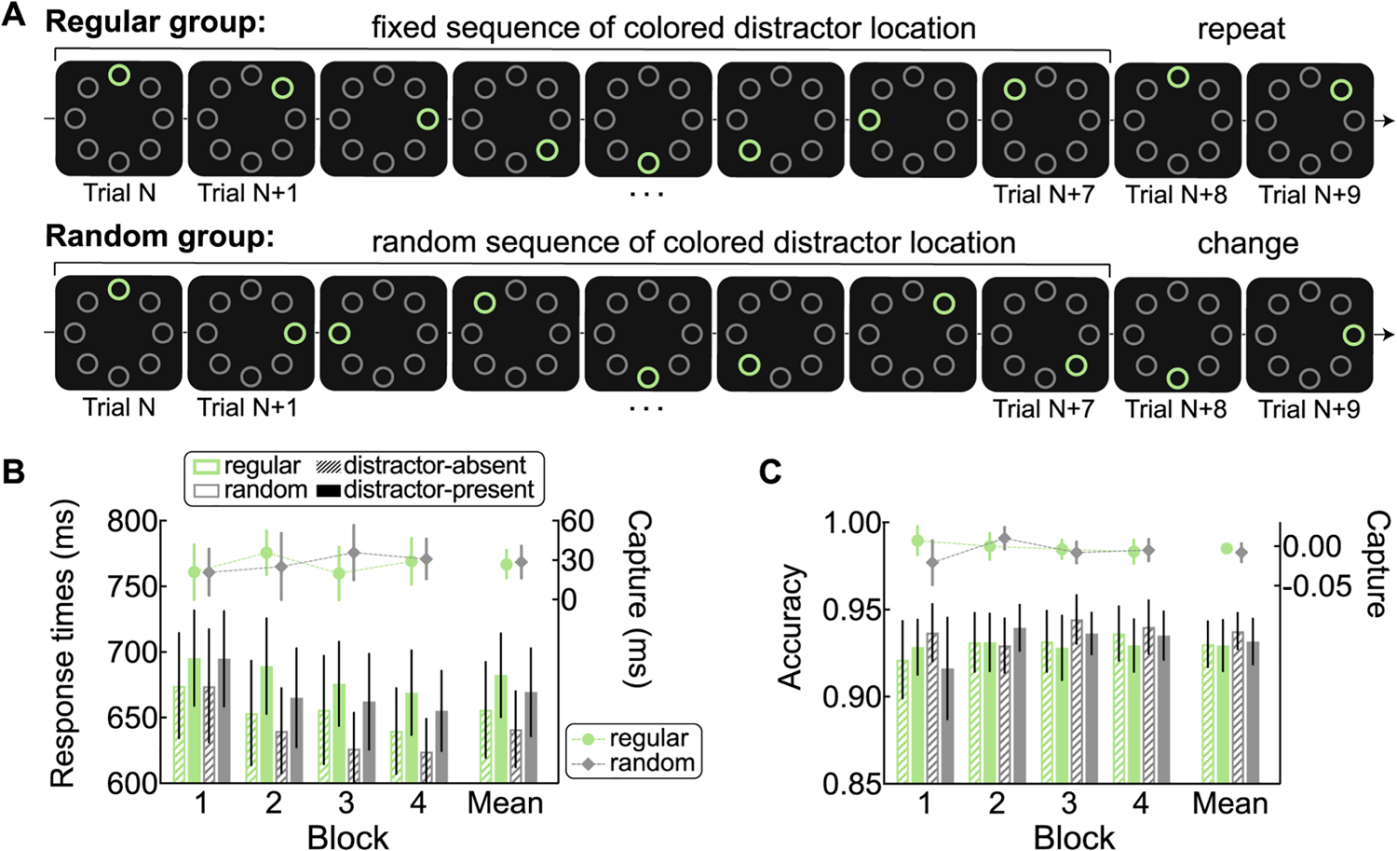
Build-in regularity pattern and results of Experiment 5. **A** Example of the sequence regarding the colored distractor location (the green circle) for the regular group and random group. Unlike the four previous experiments, for each participant, the target shape was fixed throughout all blocks; the target color was always grey while the color of the salient distractor (red or green) swapped randomly from trial to trial. **B** RTs (left *y*-axis) as a function of distractor condition (distractor-absent and distractor-present) and corresponding capture effect (distractor-present – distractor-absent, right *y*-axis) in each block for the regular group and random group. **C** Accuracies (left *y*-axis) as a function of distractor condition and corresponding capture effect (right *y*-axis) in each block for the regular group and random group. The error bars indicate 95% confidence intervals. (Color figure online)

Figure 5B and C show the RTs and accuracies as a function of distractor presence and the calculated capture effect (distractor-present – distractor-absent) in each block for the regular group and random group. Mean RTs were submitted into a three-way mixed ANOVA with block and distractor presence as within-subjects factors and with group as between-subjects factor. The main effect of distractor presence was significant, *F*(1, 78) = 46.54, *p*_c_ < .001, η_p_^2^ = 0.37, but not the Group × Distractor Presence interaction, *F*(1, 78) = 0.05, *p* = .83, η_p_^2^ = 0.00, BF_01_ = 7.86 (moderate evidence for the absence of the interaction). The main effect of block was significant, *F*(3, 234) = 14.93, *p*_c_ < .001, η_p_^2^ = 0.16, but not the Group × Block interaction, *F*(3, 234) = 1.27, *p*_c_ = .29, η_p_^2^ = 0.02, BF_01_ = 10.92. Neither the Block × Distractor Presence interaction, *F*(3, 234) = 0.48, *p*_c_ = .68, η_p_^2^ = 0.01, BF_01_ = 39.8, nor the Group × Block × Distractor Presence interaction, *F*(3, 324) = 0.72, *p* = .53, η_p_^2^ = 0.01, BF_01_ = 22.34, was significant, showing strong evidence for the absence of the interaction. For each block, independent-samples *t* tests, comparing RTs in each distractor presence condition between the two groups showed no effects (all *p*s > .23). Similarly, for each block, there was no difference in capture between the two groups (all *p*s > .27). These results indicate that the regularity did not affect the size of the capture effect.

The three-way ANOVA on accuracies did not show any significant effect (all *p*s > .1), so we only report here the values of the most important effects, including the insignificant Group × Distractor Presence interaction, *F*(1, 78) = 0.58, *p* = .45, η_p_^2^ = 0.01, BF_01_ = 6.08, and the insignificant Group × Block × Distractor Presence interaction, *F*(1, 78) = 2.04, *p* = .11, η_p_^2^ = 0.03, BF_01_ = 4.13.

#### Across-trial distance of distractor location

In this control analysis, for distractor-present trials in the random group, we restricted the across-trial distractor distance to one. A mixed ANOVA, with the within-subjects factor distractor condition (distractor-absent vs. distractor-present) and the between-subjects factor group (regular vs. random) was conducted on mean RTs. Neither the main effect of group, *F*(1, 78) = 0.55, *p* = .46, η_p_^2^ = 0.01, BF_01_ = 1.56, nor the interaction effect, *F*(1, 78) = 0.15, *p* = .70, η_p_^2^ = 0.00, BF_01_ = 4.28, was significant.

#### Awareness of the regularities

Eight out of 40 participants reported to have been aware of the across-trial association of the salient distractor location during the experiment (CS: 3.5). Two of them correctly chose all predicted locations (CS: 4.5) while six of them correctly chose none of the predicted locations (CS: 1.94). The remaining 34 participants reported to be unaware of the across-trial distractor location regularities (CS: 3.65). The mean CS regarding locations they chose was 2.04.

### Discussion

This experiment was specifically designed with the aim to replicate the findings reported by Wang et al. (2021). The results are clear in that we were not able to replicate their findings as there was no hint of a difference in attentional capture between the group of participants exposed to the regularity versus the group exposed to the random condition. At this point it is not clear why the result of Wang et al. (2021) could not be replicated. It is worth noting that our sample size was substantially larger (40 per group in our replication experiment versus 24 per group in the original experiment). Moreover, the across-trial distractor learning that was observed in the Wang et al. (2021) cannot be attributed to the awareness of the regularity by participants. Note that 20% of participants in our replication experiment, and none of the participants in the original Wang et al. (2021) reported aware of the regularities. The Bayes factors for the critical Group × Distractor Presence and Group × Block × Distractor Presence interactions revealed that our nonsignificant results do not simply indicate data insensitivity (Dienes, 2014), rather they indicate substantial to strong evidence for the lack of a learning effect. The failure to replicate Wang et al. (2021) is consistent with the study of H. Yu et al. (2022) who also tried a direct replication and failed to find the effect reported by Wang et al. (2021).

Importantly, for the current discussion, the current findings are fully consistent with the overall findings of the present study in that participants are not able to learn across-trial distractor location regularities, not even if they are as simple as in the last experiment. Unlike learning across-trial target location regularities (Li et al., 2022; Li & Theeuwes, 2020), the current study shows that participants do not learn spatial across-trial distractor regularities.

## General discussion

Multiple of recent studies have demonstrated that observers are sensitive to across-trial regularities regarding target locations, as search improved for trials in which the upcoming target location was predicted by the previous trial(s) (Boettcher et al., 2022; Li et al., 2022; Li & Theeuwes, 2020; Toh et al., 2021). Yet the current study asked the question whether participants are also able to learn these across-trial regularities when these regularities concern the distractor location which are considered not directly relevant for the task at hand. In five different experiments, participants searched for a shape singleton while ignoring the task-irrelevant distractor color singleton. In Experiments 1 and 2, paired regularities were used, which meant the distractor location on the preceding trial predicted the distractor location on the following trial. If observers would have been sensitive to the embedded location pairs, this would have resulted in faster search times for the second trial in a pair as here the distractor location is fully predictable. Yet we observed no difference in search performance between predictable and unpredictable trials. Contrary to previous findings for pairs of target locations, regular pairs of distractor locations did not facilitate search. In Experiments 3–5, the regularities were extended to longer sequences: all possible distractor locations were now presented in a structured order which was repeated throughout the whole experiment. Search performance of these groups exposed to structured orders were compared with that of another group of participants for whom the distractor location was random. We again observed no difference in raw RTs/accuracies or in the corresponding capture effect between two groups. Across five experiments, we demonstrated that participants were unable to make use of the spatial across-trial distractor regularities to suppress an upcoming distractor location as to reduce the interference it causes.

Our findings point to an important boundary condition for the modulation of visual attention and highlight the need to differentiate between distributional regularities and transitional regularities, a distinction that has also been made in the classical statistical learning literature by accounts that consider this type of learning a componential ability that spans several separable dimensions (Bogaerts, Siegelman, et al., 2022a; Growns et al., 2020; Siegelman et al., 2017). Both a simple distributional target location regularity (i.e., a certain location contains the target more frequently) and transitional across-trial regularities (i.e., a certain target location on trial *N* predicts the target location on *N* + 1) induce an implicit attentional bias that facilitates search (e.g., Geng & Behrman, 2005; Li & Theeuwes, 2020). The literature on learned location-based suppression of distractors induced by spatial statistical regularities suggests that there is robust learning of a simple distributional distractor regularity (e.g., Ferrante et al., 2018; Wang & Theeuwes, 2018b) and a recent study by Wang et al. (2021) provided initial evidence for the learning of transitional across-trial distractor locations, leading to across-trial spatial suppression. Yet the current five experiments all suggest there is basically no evidence for the learning of transitional spatial distractor regularities, either pairs or longer sequences, in visual search. Interestingly, a similar dissociation between learned suppression based on a distributional regularity and the lack thereof with across-trial predictability has been observed when manipulating expectations of color singletons’ occurrence: reduced attentional capture was observed in environments characterized by a high likelihood of distractors (even when their location and features are unpredictable; Won et al., 2019) but not by distractor presence that could be anticipated on a trial-by-trial basis (Bogaerts, van Moorselaar, & Theeuwes, 2022b).

The current Experiments 1 and 2 used the same pairs of spatial locations as Li and Theeuwes (2020) who examined across-trial target regularities. Despite the overlap in the spatial pattern and the fact that in all studies the across-trial regularities involved salient singletons, Li and Theeuwes (2020) observed search performance benefits on predicted trials, while the present study did not. There may be several reasons why learning across-trial regularities regarding the target is possible while learning across-trial regularities regarding distractor is not. First, while attention is inevitably captured by the salient distractor singleton (especially in Experiment 2 in which capture was very large), attention is likely to be shifted away from the distractor immediately resulting in relatively short attentional dwelling at the location of the distractor (e.g., Born et al., 2011; Theeuwes et al., 1998; Wang et al., 2019). Because attention is so briefly at the location of the distractor, learning of across-trial distractor-distractor associations might be more difficult, if possible at all. Clearly the distractor is in principle task-irrelevant and following initial capture by the distractor, attention is immediately reshifted to the location of the target. This implies that after (briefly) attending the distractor location, within the same search trial, there is another shift of attention towards the target location. Considering that the target location is irrelevant to the across-trial distractor regularity, but it is attended in between the distractor locations that make up the regularity, this may hamper the formation of distractor–distractor associations. How does this compare to the situation of across-trial target–target associations where learning does take place (Li & Theeuwes, 2020)? A target is of course task-relevant and attention dwells at the location of the target for a relatively long time as participants have to determine the orientation of the line segment inside the element to be able to give the correct response. The presence of a color singleton distractor that captures attention can also introduce a situation in which an irrelevant distractor location intervenes in the sense that it is shortly attended between the two target locations whose transition is predictable. Hence, it is worth noting that in Li and Theeuwes (2020) the extraction of the across-trial target–target associations was not affected by the presence of the color singleton distractor in the search display.

In Experiments 3–5 we pushed the limits of distractor learning further by presenting participants with a structured sequence of distractor locations. These should be easier to learn compared with “pairs among randomness” as used in Experiments 1 and 2. Yet neither experiment showed any hint that the sequential spatial regularities were learned and could be used to reduce attentional capture. Overall, our findings are consistent with previous studies demonstrating that the ability of observers to learn and utilize the across-trial regularities in complex environments is limited, even if it is the target location that was predicted (Bouwkamp et al., 2021; Li et al., 2022; Ono et al., 2005; Thomas et al., 2018; Toh et al., 2021). For example, although Li and Theeuwes (2020) found faster RTs on predicted trials, it is noteworthy that such benefits were only observed in pop-out feature search, but not in slow serial search such as in a *T*-among-*L*s serial search task (Li et al., 2022). A similar finding was observed when the target location was presented in a repeating 12-item sequence: the learning (which was expressed as slower RTs when the sequence was disrupted) was present in a single target condition or during pop-out feature search but absent during serial search (Toh et al., 2021). Using the contextual cueing paradigm, Ono et al. (2005) found that repeating the target or distractors configurations even when they were 100% predictive of the following target location was insufficient to form across-trial spatial associations. Across-trial contextual cueing only occurred when a repeated target–distractor configuration was predictive of the following target location. Bouwkamp et al. (2021) who extended the Ono et al. (2005) study by presenting the repeated search displays in a structured order or in a random order (within-subjects design) found that performance on the sequential search scenes (i.e., spatially predictive) was not better than on the randomized-order ones. Boundary conditions for learning have also been identified when varying the temporal gap between contingencies. Thomas et al. (2018) found that such across-trial contextual cueing was confined to temporally close contingencies (i.e., the predicted target location on trial *N* can be learned by the repeated configuration of trial *N* − 1 but not trial *N* − 2). Taken together, these findings suggest that even for task-relevant targets, the learning of transitional spatial regularities (rather than a static high-probability location) might be relatively fragile.

No benefits in performance were observed when introducing across-trial spatial regularities in the search display, but at this point it is unclear whether participants did not learn the distractor spatial regularities, or rather they learned these but were not able to apply their (implicit) knowledge of the regularities to reduce attentional capture. The above discussion suggests that there is no across-trial distractor location learning. However, one may argue that participants learned but did not use the regularity. Specifically, learning such across-trial distractor regularity could only help to rule out one impossible target location, with seven remaining possible target locations. In this case, using the regularity may contribute little to facilitating search. However, previous research using explicit cues to indicate the upcoming distractor location suggests that the reduced interference by the distractor was not due to the target activation of uncued locations but instead due to the suppression of the cued location (e.g., Chao, 2010; Munneke et al., 2008). Also, when participants were told that the upcoming distractor was salient, they appeared to be able to prepare in advance to reduce the capture (Heuer & Schubö, 2019; Munneke et al., 2011; Ruff & Driver, 2006; van Zoest et al., 2021). Note, however, a study by Wang and Theeuwes (2018a) demonstrated that cuing the upcoming salient distractor location on a trial-to-trial basis does not result in proactive spatial suppression, which might explain our null findings across five experiments.

It is also possible that the regularity was used but benefits were not expressed at the behavioral level, as was also observed in recent work by Heuer and Schubö (2019). In this study participants’ electroencephalogram signals were recorded. Before the search array, a distractor cue was presented, either predictive of the upcoming distractor location with 100% validity (valid cue) or predictive nothing (neutral cue). Heuer and Schubö (2019) did not find any behavioral benefits between valid cue and neutral cue conditions. However, compared with when the cue was unpredictive, they observed that the salient distractor following the valid cue elicited a smaller P_D_ component which is related to active distractor suppression. In addition, there was no difference between two conditions in the N_T_ component which is a marker of target activation. Heuer and Schubö (2019) claimed that the predictable distractor locations were processed anticipatorily so that when the distractor was presented, the inhibitory processing needed was reduced. Therefore, our findings do not necessarily imply that the temporal predictive context was not processed. Nevertheless, it is clear that, proactive spatial suppression elicited by across-trial SL of distractor locations might be difficult or even impossible.

The current set of experiments focused on transitional regularities regarding the spatial location of distractors. Our findings showing a lack of across-trial learning therefore refers to a lack of spatial distractor suppression. In the first two experiments the color of the distractor swapped randomly across trials, making it possible to test whether repeating the feature of the distractor affected across-trial learning. We found an overall effect of feature repetition; yet this effect did not contribute to across-trial learning of the spatial distractor regularity. However, our findings do not preclude that across-trial distractor learning is possible if the regularity is about the feature of the distractor (its color or shape) instead of its location. Previous research has demonstrated learned suppression in the nonspatial domain (see Chelazzi et al., 2019; Geng et al., 2019; van Moorselaar & Slagter, 2020, for recent reviews), for example, distractor colors that appear with a high probability are suppressed more efficiently compared with low-probability colors (Failing et al., 2019). However, the research focus has been on distributional regularities and to our knowledge there is to date no work that examined the learning of distractor features that are predictable on a trial-by-trial basis. The learnability of nonspatial across-trial distractor regularities thus remains to be explored in future research.

In conclusion, even though previous studies demonstrated across-trial VSL results in attentional biases that benefit search performance, the current five experiments indicate no evidence of across-trial distractor-distractor associations driving proactive spatial suppression. The present study provides important boundary conditions for the modulation of attention by statistical regularities that concern the location of task-irrelevant distractors.
